# Supplementary material for: Mapping the evolution of fertility support policies in China: A content and instrumental analysis
Source: PLoS One. 2025 Oct 9;20(10):e0332137. doi: 10.1371/journal.pone.0332137 (PMC12510515; doi:10.1371/journal.pone.0332137)
Supplement: S1 Appendix — (ZIP) [file pone.0332137.s001.zip › S1 Appendix. 226 original policy documents/53-国务院办公厅关于进一步加大对中小企业纾困帮扶力度的通知(FBM-CLI-2-5078871).doc]

国务院办公厅关于进一步加大对中小企业纾困帮扶力度的通知

**发布部门：** [国务院办公厅](https://www.pkulaw.com/law/chl?Aggs.IssueDepartment=601) [机构沿革](javascript:void(0))

**发文字号：**国办发〔2021〕45号

**发布日期：**2021.11.10

**实施日期：**2021.11.10

**时效性：** [现行有效](https://www.pkulaw.com/law/chl?Aggs.TimelinessDic=01)

**效力级别：** [国务院规范性文件](https://www.pkulaw.com/law/chl?Aggs.EffectivenessDic=XC0203)

**法规类别：** [市场管理](https://www.pkulaw.com/law/chl?Aggs.Category=08204) [企业综合规定](https://www.pkulaw.com/law/chl?Aggs.Category=04501)

国务院办公厅关于进一步加大对中小企业纾困帮扶力度的通知
（国办发〔2021〕45号）

各省、自治区、直辖市人民政府，国务院各部委、各直属机构：
　　中小企业是国民经济和社会发展的主力军，在促进增长、保障就业、活跃市场、改善民生等方面发挥着重要作用。近期，受原材料价格上涨、订单不足、用工难用工贵、应收账款回款慢、物流成本高以及新冠肺炎疫情散发、部分地区停电限电等影响，中小企业成本压力加大、经营困难加剧。为贯彻落实党中央、国务院决策部署，进一步加大助企纾困力度，减轻企业负担，帮助渡过难关，经国务院同意，现就有关事项通知如下：
　　**一、**加大纾困资金支持力度。鼓励地方安排中小企业纾困资金，对生产经营暂时面临困难但产品有市场、项目有前景、技术有竞争力的中小企业，以及劳动力密集、社会效益高的民生领域服务型中小企业（如养老托育机构等）给予专项资金支持，减轻房屋租金、水电费等负担，给予社保补贴等，帮助企业应对原材料价格上涨、物流及人力成本上升等压力。落实创业担保贷款贴息及奖补政策。用好小微企业融资担保降费奖补资金，支持扩大小微企业融资担保业务规模，降低融资担保成本。有条件的地方要发挥好贷款风险补偿机制作用。（财政部、工业和信息化部、人力资源社会保障部、人民银行等国务院相关部门及各地区按职责分工负责）

　　**二、**进一步推进减税降费。深入落实月销售额15万元以下的小规模纳税人免征增值税、小型微利企业减征所得税、研发费用加计扣除、固定资产加速折旧、支持科技创新进口等税收优惠政策。制造业中小微企业按规定延缓缴纳2021年第四季度部分税费。研究适时出台部分惠企政策到期后的接续政策。持续清理规范涉企收费，确保政策红利落地。（财政部、税务总局、海关总署、市场监管总局等国务院相关部门及各地区按职责分工负责）

　　**三、**灵活精准运用多种金融政策工具。加强再贷款再贴现政策工具精准“滴灌”中小企业，用好新增3000亿元支小再贷款额度。加大信用贷款投放，按规定实施普惠小微企业信用贷款支持政策。对于受新冠肺炎疫情、洪涝灾害及原材料价格上涨等影响严重的小微企业，加强流动资金贷款支持，按规定实施普惠小微企业贷款延期还本付息政策。（人民银行、银保监会按职责分工负责）

　　**四、**推动缓解成本上涨压力。加强大宗商品监测预警，强化市场供需调节，严厉打击囤积居奇、哄抬价格等违法行为。支持行业协会、大型企业搭建重点行业产业链供需对接平台，加强原材料保供对接服务。推动期货公司为中小企业提供风险管理服务，助力中小企业运用期货套期保值工具应对原材料价格大幅波动风险。稳定班轮公司在中国主要出口航线的运力供给。发挥行业协会、商会及地方政府作用，引导外贸企业与班轮公司签订长约合同，鼓励班轮公司推出中小企业专线服务。（国家发展改革委、工业和信息化部、市场监管总局、中国证监会、交通运输部、商务部等国务院相关部门及各地区按职责分工负责）

　　**五、**加强用电保障。加强电力产供储销体系建设，科学实施有序用电，合理安排错峰用电，保障对中小企业尤其是制造业中小企业的能源安全稳定供应。推动产业链龙头企业梳理上下游重点企业名单，保障产业链关键环节中小企业用电需求，维护产业链供应链安全稳定，确保企业已有订单正常生产，防范订单违约风险。加快推进电力市场化改革，充分考虑改革进程和中小企业承受能力，平稳有序推动中小企业进入电力市场。鼓励有条件的地方对小微企业用电实行阶段性优惠。（国家发展改革委、工业和信息化部及各地区按职责分工负责）

　　**六、**支持企业稳岗扩岗。落实失业保险稳岗返还及社保补贴、培训补贴等减负稳岗扩就业政策，支持中小企业稳定岗位，更多吸纳高校毕业生等重点群体就业。推动各级政府公共服务平台、人力资源服务机构为中小企业发布实时有效的岗位信息，加强用工供需信息对接。（人力资源社会保障部、财政部及各地区按职责分工负责）

　　**七、**保障中小企业款项支付。进一步落实《[保障中小企业款项支付条例](https://www.pkulaw.com/chl/9c0d4000d319931ebdfb.html)》，制定保障中小企业款项支付投诉处理办法，加强大型企业应付账款管理，对滥用市场优势地位逾期占用、恶意拖欠中小企业账款行为，加大联合惩戒力度。继续开展清理拖欠中小企业账款专项行动。推动各级政府部门、事业单位、大型企业及时支付采购中小企业货物、工程、服务的账款，从源头防范层层拖欠形成“三角债”。严禁以不签合同、在合同中不约定具体付款时限和付款方式等方法规避及时支付义务的行为。（工业和信息化部、国务院国资委、财政部、人民银行等国务院相关部门及各地区按职责分工负责）

　　**八、**着力扩大市场需求。加大民生领域和新型基础设施建设投资力度，进一步落实《政府采购促进中小企业发展管理办法》，鼓励各地因地制宜细化预留采购份额、价格评审优惠、降低投标成本、优先采购等支持措施。组织开展供需对接活动，促进大型企业扩大向中小企业采购规模。搭建政银合作平台，开展中小企业跨境撮合服务。依托跨境电商等外贸新业态，为中小企业提供远程网上交流、供需信息对接等服务。加快海外仓发展，保障外贸产业链供应链畅通运转。充分发挥境外经贸合作区作为中小企业“抱团出海”平台载体的作用，不断提升合作区建设质量和服务水平，引导和支持有合作需求的中小企业入区开展投资合作。（国家发展改革委、财政部、商务部、工业和信息化部按职责分工负责）

　　**九、**全面压实责任。各有关部门、各地区要进一步把思想认识行动统一到党中央、国务院决策部署上来，强化责任担当，勇于开拓创新，进一步细化纾困举措，积极采取针对性措施，帮助中小企业应对困难，推动中小企业向“专精特新”方向发展，不断提升市场竞争力。各有关部门要加强对中小企业面临困难和问题的调研，总结经验做法，加强政策储备，适时推动出台；要加大对地方的指导支持力度，扎实推动各项政策措施落地见效。落实情况要及时报送国务院促进中小企业发展工作领导小组办公室。（国务院相关部门及各地区按职责分工负责）

国务院办公厅
　　2021年11月10日

© 北大法宝：（[www.pkulaw.com](https://www.pkulaw.com/)) 专业提供法律信息、法学知识和法律软件领域各类解决方案。北大法宝为您提供丰富的参考资料，正式引用法规条文时请与标准文本核对。
欢迎查看所有[产品和服务](https://www.pkulaw.com/helps/2.html)。法宝快讯：[如何快速找到您需要的检索结果？](https://www.pkulaw.com/helps/70.html)[法宝](https://www.pkulaw.com/helps/69.html)V6有何新特色？

扫描二维码阅读原文

**原文链接：**<https://www.pkulaw.com/chl/c618c3c181a26bcfbdfb.html>
